# Artificial intelligence powers digital medicine

**DOI:** 10.1038/s41746-017-0012-2

**Published:** 2018-03-14

**Authors:** Alexander L. Fogel, Joseph C. Kvedar

**Affiliations:** 1 Stanford University School of Medicine, Stanford, CA USA; 2 Department of Dermatology, Massachusetts General Hospital, Boston, MA USA; 3 Harvard Medical School, Boston, MA USA; 4 Partners Connected Health, Partners Healthcare, Boston, MA USA

**Keywords:** Epidemiology, Epidemiology

## Abstract

Artificial intelligence (AI) has recently surpassed human performance in several domains, and there is great hope that in healthcare, AI may allow for better prevention, detection, diagnosis, and treatment of disease. While many fear that AI will disrupt jobs and the physician–patient relationship, we believe that AI can eliminate many repetitive tasks to clear the way for human-to-human bonding and the application of emotional intelligence and judgment. We review several recent studies of AI applications in healthcare that provide a view of a future where healthcare delivery is a more unified, human experience.

## Introduction

Artificial intelligence (AI) powers the digital age. While this reality has become more tangible in recent years through consumer technology, such as Amazon’s Alexa or Apple’s Siri, the applications of AI software are already widespread, ranging from credit card fraud detection at VISA to payload scheduling operations at NASA to insider trading surveillance on the NASDAQ. Broadly defined as the imitation of human cognition by a machine, recent interest in AI has been driven by advances in machine learning, in which computer algorithms learn from data without human direction.^1^ Most sophisticated processes that involve some form of prediction generated from a large data set use this type of AI, including image recognition, web-search, speech-to-text language processing, and e-commerce product recommendations.^2^ AI is increasingly incorporated into devices that consumers keep with them at all times, such as smartphones, and powers consumer technologies on the horizon, such as self-driving cars. And there is anticipation that these advances will continue to accelerate: a recent survey of leading AI researchers predicted that, within the next 10 years, AI will outperform humans in transcribing speech, translating languages, and driving a truck.^3^

Despite a flurry of recent discussion about the role and meaning of AI in medicine, in 2017 nearly 100% of U.S. healthcare will be delivered with 0% AI involvement. In healthcare, there is great hope that AI may enable better disease surveillance, facilitate early detection, allow for improved diagnosis, uncover novel treatments, and create an era of truly personalized medicine. There is also profound fear on the part of some that it will overtake jobs and disrupt the physician–patient relationship, e.g., AI researchers predict that AI-powered technologies will outperform humans at surgery by 2053.^3^ The wealth of data now available in the form of clinical and pathological images, continuous biometric data, and internet of things (IoT) devices are ideally suited to power the deep learning computer algorithms that lead to AI-generated analysis and predictions. Consequently, there has been a substantial increase in AI research in medicine in recent years.

We believe, based on several recent early-stage studies, that AI can obviate repetitive tasks to clear the way for human-to-human bonding and the application of emotional intelligence and judgment in healthcare. Physician time is increasingly limited as the number of items to discuss per clinical visit has vastly outpaced the time allotted per visit,^4^ as well as due to the increased time burden of documentation and inefficient technology.^5^ Given the time limitations of a physician’s, as the time demands for rote tasks increase, the time for physicians to apply truly human skills decreases. By embracing AI, we believe that humans in healthcare can increase time spent on uniquely human skills: building relationships, exercising empathy, and using human judgment to guide and advise.

## Black box warning

AI has already exceeded human performance in visual tasks,^6^ large-scale image recognition,^7^ and strategy games^8^ due to rapid advances in the field of deep learning.^9^ Previously, machine-driven predictions relied on algorithms designed to extract specific features provided by a human expert. For example, the designer of a melanoma detection program might input rules that detect asymmetry and border irregularity. First-gen machines were limited in their accuracy by relying only on rules that could be programmed, and unless new rules were specifically added, the machines were unable to adapt. Now, the advent of deep learning algorithms allows for machines to receive data and self-develop complex functions to provide predictions. Using the melanoma example, now the designer of our melanoma detection program simply feeds the computer labeled images of confirmed melanomas and non-melanomas, and the computer creates its own internal rules to differentiate malignant from benign. And as the machine collects more data, it can continue to improve its predictions.

Current AI therefore creates an uncomfortable situation for physicians and patients: we cannot tell which features the machine uses to generate its predictions. Without a thorough understanding of how AI is working, it may be difficult to assuage the fear of “runway machines” that has been stoked by movies like *The Matrix*, *The Terminator*, and *2001: A Space Odyssey*. There has accordingly been significant discussion on the ethical implementation of AI.^10^

Regardless, it will quickly become clear that AI can equal or outperform humans at simple, repetitive tasks. And the simpler or more helpful the task—for example an AI system that can largely automate electronic medical record documentation—the easier it will be to allow these technologies into the clinic. Physicians by and large don’t enjoy repetitive, rote tasks—they enjoy the application of reason and judgment to complex problems in order to help patients. Rather than take over, we believe that these systems may take on much of the unpleasant work of healthcare.

### A lens into the future

Much of the recent interest in AI-enabled medical care comes from other fields, such as consumer products, or activities in healthcare that do not involve patient care, such as marketing. Studies of AI in healthcare are early: most are pre-clinical, small, or test AI technology in artificial environments that would be difficult to replicate in real-world clinical settings. However, these studies provide a lens into the future of how AI technologies might be incorporated clinically. Because of its ability to collect and analyze vast quantities of data, as well as the greater speed at which diagnosis will occur, we believe that AI enables a less fragmented, more human experience. While rigorous validation and clinical testing is still needed for all AI technologies in healthcare, here we describe several of the most promising studies.

### Skin cancer screening

Skin cancers are the most common human cancers, totaling 5.4 million new cases and more than 10,000 deaths each year in the U.S.^11^ As visible malignancies, early detection is not only possible but critical: for example, melanoma 5-year survival drops from more than 99 to 14% based on earliest vs. latest stage detection.^12^ Performing a skin exam to check for cancer is a difficult and time-consuming task, and requires dermatologist expertize that is already in significant shortage.^13^

In 2017, Esteva and Colleagues created a deep convoluted neural network that was able to differentiate images of malignant and benign skin lesions with performance comparable to a panel of board-certified U.S. dermatologists.^12^ The authors trained their neural network with a dataset of hundreds of thousands of images from physician-curated open access dermatology datasets and data from Stanford Hospital. After training, the computer was tested against 21 dermatologists on images of skin lesions for pathology-confirmed melanoma and non-melanoma skin cancer, as well as dermatoscopic images of pathology-conformed melanoma. In a test of sensitivity and specificity, the computer outperformed the average dermatologist in the test, and generated AUC values between 0.91 and 0.96.

While significantly more work remains to be done in terms of clinical validation, the implications of this technology’s ability to aid dermatologists, augment the scope of primary care practice, and expand access to care in regions without access to dermatologist-level skin cancer screenings are profound. While this technology was deployed on specially prepared images rather than real-life clinical settings, one can imagine its use on mobile devices or in clinical settings in the near future. By reducing the time to perform a clinical visit, future iterations of this technology may enable dermatologists to spend even more time with patients to develop human-to-human bonds of trust, and using judgment to create treatment and prevention plans that match patient goals.

### Diabetic retinopathy

Diabetes affects 29.1 million Americans, and the cost of caring for these individuals is $245 billion annually.^14^ Given that another 86 million Americans have pre-diabetes, which confers a high likelihood of diabetes development in the future, there is significant concern as to how to manage this growing epidemic.^14^ Eye care is critical, as 28.5% of U.S. diabetics have diabetic retinopathy, which can lead to blindness.^15^ Screening involves a dilated eye exam 1–2 times annually, with referral to an ophthalmologist if retinopathy is graded as moderate or worse or if macular edema is observed.^16^ While in-person dilated eye exams are performed, retinal photography with manual interpretation is a well-established screening method.

In 2016, Gulshan et al. developed a deep neural network to evaluate images for diabetic retinopathy.^17^ The computer was trained using 128,175 images previously evaluated by a panel of 54 board-certified U.S. ophthalmologists and senior ophthalmology residents. It was then tested on two data sets consisting of 9963 and 1748 images previously classified by 7 board-certified U.S. ophthalmologists with highest rates of self-consistency from the previous panel. The computer achieved an AUC of 0.97–0.99 for detecting referable diabetic retinopathy.

Given that much of diabetic retinopathy screening is already performed on images reviewed remotely, this technology has the potential to increase the speed and accuracy of retinopathy screening to the point-of-care, and may allow for increased access to care as well as early detection and treatment. However, the computer was not tested against human retinopathy screeners and did not evaluate for features of other diseases, such as macular degeneration or glaucoma, that are assessed by manual retinopathy screening programs.

While the use of this technology appears simply to eliminate human work, it appears to us more likely that a future with automated retinopathy screening will re-direct the work of human eye specialists from grading images to managing the greater volume of person-to-person care produced by more widespread screening. The application of human judgment to help diabetic patients improve their eye health—the ultimate goal of screening for diabetic retinopathy—will become a more critical task for humans to perform.

### Medication adherence

Medication adherence is a significant issue given concerns about healthcare outcomes and rising healthcare costs. Medication nonadherence contributes to 125,000 deaths accounts for more than $100 billion in healthcare costs each year.^18–20^ Research indicates that about half of prescriptions are not taken as directed and 20–30% are never filled.^18^

A major goal of the digital medicine community is to increase medication adherence. In 2017, Labovitz et al. used a smartphone deployment of an AI platform to measure adherence in patients on direct oral anticoagulants.^21^ While this class of medications has reduced the frequency of laboratory monitoring needed for earlier generations of anticoagulants, such as warfarin, they have placed more self-monitoring burden on patients, and it is more difficult for physicians to detect suboptimal adherence. The authors used a neural network computer vision algorithm using the smartphone’s camera to visually identify the patient, the drug and confirm ingestion, and then correlated these results with pill counts and plasma sampling in both the AI monitoring and unmonitored control group. Over the course of 12 weeks in 28 patients, the authors found that adherence was 100% in the intervention group compared with 50% in the control group. Results from a larger, more robust studies are needed to determine whether this substantial improvement in adherence can be maintained.

Furthermore, adherence is a critical part of clinical trials, which currently use a combination of indirect measures such as pill counts and self-reported data that are known to contain inaccuracies and biases. In a 2017 study, Bain and Colleagues assessed the use of an AI platform deployed on a smartphone to assess adherence in a phase II clinical trial of the α7 nicotinic receptor agonist (ABT-126) in patients with schizophrenia.^22^ Their platform used a neural network computer vision algorithm using the smartphone’s camera to visually identify the patient, the drug and confirm ingestion. The results were then correlated with pharmacokinetic adherence data for 24 weeks. Adherence in the AI group was 17.9% higher than standard-of-care modified directly observed therapy protocol. The study also found that the AI platform used was able to detect non-adherence more quickly and better predict future non-adherence than were conventional methods. Though these results are from a non-randomized small sample (*n* = 53), it is easy to imagine AI could be deployed to assess medication usage in a broad range of settings, ranging from opted-in patient homes to clinical trials to infectious disease treatment protocols.

Currently, ascertaining medication adherence and the components that affect it is difficult for physicians: a recommendation for a patient goes in, many changing variables affect the process, and the output is typically a patient’s subjective estimate of adherence. With an AI-enabled accurate understanding of a patient’s medication adherence, a physician can spend more with the patient getting to the heart of issues that affect the patient’s individual adherence. Using judgment and emotional intelligence, the physician and patient can then develop strategies that work within the patient’s life to optimize adherence.

## AI-enabled humans

While there are many details to be worked out as AI enters healthcare—whether robust studies determine that AI-enabled care is safe and effective, who is allowed to use or interpret the technology, how AI integrates into evolving clinical care models, whether machine-generated results need to be confirmed by a physician, how will the technology fit into reimbursement systems, and who is at fault in the case of errors to name a few—the above examples hint at a future where healthcare delivery is a more seamless, personalized experience. Our hope is that this future will also allow physicians to focus on what led most of us to the field in the first place: focusing our work on other human beings, understanding their circumstances, developing bonds, and serving as a trusted advisor.

With these considerations in mind, we recommend the following in thinking about AI in healthcare:

- Patient care comes first. It seems obvious, but the primary question in thinking about AI should be “will this improve the health of patients”. Patient care is the paramount goal in healthcare, and is more important than any other consideration, including income, jobs, stability, control, etc. But if a smart machine can achieve longer, healthier lives for patients, it should, and by enabling it to do so you are helping your patients. Don’t end up on the wrong side of history by fighting against improvements in patient outcomes.
- Embrace change. Working with, rather than against, technology will best enable it to develop in ways that are complementary to our fields. Each generation for the last 100 years has had a visceral fear that automation would replace jobs. And yet, as it does so, new jobs emerge. In healthcare, this may mean radical changes to existing fields.^23^ Active participants in the evolution of AI by stakeholders—patients, physicians, researchers, government and industry—is the best way to ensure these technologies are working to improve patient care, streamline clinical workflow, and enhance the healthcare experience.
- And especially for physicians: invest in your human abilities. Honestly evaluate the strengths and weaknesses in your interpersonal skills and judgment. Can you read people’s emotions? Do you easily develop trust with others? How good are you at motiving people? Medical schools, residency programs, and continuing medical education should look closely at the curriculum of business schools, which teach evidence-based approaches for honing interpersonal skills.^24^ Training and re-training will be critical. Ultimately, individual physicians will need to develop strategies to better empathize, relate to, advise, influence and manage other humans.

## Conclusion

As repetitive tasks are automated, humans will be able to focus on the tasks that are uniquely human: building relationships, exercising empathy, and using human judgment to guide and advise. Perhaps AI will enable healthcare to be more human.
